# Cytomegalovirus infection in HIV-infected and uninfected individuals is characterized by circulating regulatory T cells of unconstrained antigenic specificity

**DOI:** 10.1371/journal.pone.0180691

**Published:** 2017-07-06

**Authors:** Adriana Tovar-Salazar, Adriana Weinberg

**Affiliations:** School of Medicine, University of Colorado Denver, Anschutz Medical Center, Aurora, Colorado, United States of America; IMAGINE, FRANCE

## Abstract

Cytomegalovirus (CMV) infection is associated with immune-suppression in immune-compromised hosts and old adults. We previously showed that *ex vivo* CMV restimulation of peripheral blood mononuclear cells (PBMC) of CMV-seropositive volunteers expanded CD4+CD27-CD28- regulatory T cells (Tregs). Here we evaluate the phenotype and function of circulating CD4+CD27-CD28- T cells of CMV-seropositive adults. Compared with CMV-seronegative, CMV-seropositive adults had 10-fold higher CD4+CD27-CD28-% T cells in PBMC. Circulating CD4+CD27-CD28- T cells from both CMV-seropositive and seronegative donors expressed higher levels of TGFβ, granzyme B, CD39, CD147 and IL-35, and lower levels of CD127, compared with their parent circulating CD4+ T cells. However, only CMV-seropositive circulating CD4+CD27-CD28- had increased FOXP3 expression. CD4+CD27-CD28- sorted from the PBMC of CMV-seropositive donors expanded *ex vivo* in the presence of rhIL2 and inhibited *ex vivo* proliferation of autologous PBMC restimulated with CMV, varicella-zoster virus or *C*. *albicans* antigens. CD4+CD27-CD28- sorted from CMV-seronegative PBMC did not expand in the presence of rhIL2 and did not inhibit autologous PBMC proliferation. CD3+CD27-CD28- circulating T cells (≥80% CD8+) from CMV-seropositive HIV-infected donors also inhibited *ex vivo* proliferation of autologous PBMC restimulated with CMV or HIV. These data indicate that CMV-seropositive individuals have circulating Tregs that inhibit cell-mediated immune responses to CMV and other antigens and may be contribute to an immune-suppressive effect of CMV infection. Moreover, the phenotypic similarity between circulating CD4+CD27-CD28- Tregs with differentiated effector T cells suggests that the two T-cell subsets might evolve in parallel or in sequence from the same progenitor cells in response to CMV stimulation during reactivations.

## Importance

CMV infection is associated with immune-suppression. The mechanism underlying this effect is unknown. We determined that peripheral blood CD4+CD27-CD28- T cells, which generally represent effector T cells, of CMV-seropositive adults have regulatory function, which may explain the association of CMV seropositivity, high CD4+CD28- T cell frequencies and immune-suppression. Notably, we did not find regulatory T cells among peripheral blood CD4+CD27-CD28- cells of CMV-seronegative individuals. In HIV-infected CMV-seropositive individuals with low numbers of CD4+ cells, CD3+CD27-CD28- T cells (mostly CD8+CD27-CD28-) have regulatory T-cell function. Immune-suppression of CMV-seropositive old adults has also been associated with high CD4+CD28- or CD8+CD28- T-cell numbers. We propose that the regulatory CD4+CD27-CD28- and CD8+CD27-CD28- T cells of CMV-seropositive individuals contribute to the immune suppression associated with CMV infection. The data suggest that CMV effector and regulatory T cells may evolve together and that decreasing CMV T cell stimulation might limit the generation of regulatory T cells.

## Introduction

Cytomegalovirus (CMV)-infected individuals with cell-mediated immune disorders, such as transplant recipients and human immunodeficiency virus (HIV)-infected individuals, or at the extremes of age without any age-independent immune disorders have higher morbidity and mortality than uninfected individuals [1–7]. Most reports found an association of CMV infection with increased HIV disease progression and death in HIV-infected individuals, increased bacterial and fungal superinfections in transplant recipients and decreased immune responses to vaccines and increased respiratory infection in older adults [7–9], although there have also been some exceptions [10, 11]. Overall, the data suggest that CMV has an immune suppressive effect on the host.

During active infection, CMV replicates in many cells of the immune system including monocytes, macrophages and dendritic cells and subsequently establishes latency in CD34+ myeloid progenitors. Both during active and latent infection, the virus employs immune evasion mechanisms that allow it to survive in the host by suppressing host immune responses [12]. Because of the varied array of infectious and other complications that follow CMV infection, we hypothesized that the immune evasion mechanisms induced by CMV infection are not solely suppressive of immune responses to CMV, but also hamper host immune defences against other pathogens. The hypothesis that regulatory T cells (Treg) expanded in response to a specific pathogen may be cross-reactive and impair immune defences against other pathogens is supported by several mouse studies that showed that Treg expanded by chronic infection with Friend retroviruses suppress immunity to murine CMV [13, 14].

In our previous studies, we showed that in vitro stimulation of peripheral blood mononuclear cells (PBMC) from CMV-seropositive donors increased the proportion of CD4+CD27-CD28- T cells, particularly in HIV co-infected subjects [15]. Although the CD27-CD28- phenotype has been typically associated with terminally differentiated effectors, we found that the CMV-specific CD4+CD27-CD28- T cells had Treg phenotypic markers including high expression of FOXP3 and TGFβ [16]. Compared with the total CD4+ parent T cell population in CMV-stimulated cultures, the CD4+CD27-CD28- T cells also had increased expression of granzyme B (GrB), which is a cytotoxicity mediator shared by effector T cells and Treg as well as by regulatory dendritic, myeloid suppressor and B cells [17–21]. Furthermore, when added to autologous PBMC, the CMV-stimulated CD4+CD27-CD28- Treg decreased proliferation of autologous PBMC in a dose-dependent fashion to CMV and to a lesser extent also *C*. *albicans* antigenic stimulation. In a case-control study of CMV-seropositive individuals with AIDS and very low CD4+ T cells, we showed that the risk of CMV end-organ disease was independently associated with high proportions of CMV-specific GrB-secreting PBMC (ELISPOT) and high proportion of CMV-specific CD8+CD107a+ expanded form PBMC [22], supporting the notion that CMV-specific CD8+ Treg importantly contribute to the high morbidity and poor outcome of CMV-infected hosts with AIDS. Notably, other studies also described expansions of Treg in CMV-infected transplant recipients and elderly individuals [23, 24] as well as CD4+ and CD8+ Treg expansions with another herpesvirus from the CMV family, HHV6 [25].

In this study, we expand our findings in CMV-stimulated PBMC to unstimulated freshly thawed PBMC. We report on the function and phenotype of CD4+CD27-CD28- Treg in PBMC of CMV-infected individuals, with or without HIV co-infection, and compare them to CD4+CD27-CD28- PBMC from CMV-seronegative controls. In addition, we compare the regulatory effect of circulating unstimulated peripheral blood CD4+CD27-CD28- T cells of CMV-seropositive donors with that of CD4+CD27-CD28- T cells expanded by ex-vivo stimulation of PBMC from CMV-seropositive donors.

## Materials and methods

### Specimens

Cryopreserved PBMC were obtained from 14 HIV-infected and CMV-seropositive, 14 HIV-uninfected CMV-seropositive and 8 HIV-uninfected CMV-seronegative anonymous donors. This work was reviewed and considered exempt by the Colorado Multiple Institution Review Board.

### Reagents and monoclonal antibodies

The following monoclonal antibodies (mAb) were used for phenotypic characterization and cell sorting: Anti-CD4 APC (clone RPA-T4), anti-CD27 PE (clone M-T271), anti-CD28 FITC (clone CD28.2), anti-CD28 PE (clone CD28.2), anti-IL-10 APC (clone JES3-19F1), anti-Granzyme B FITC (clone GB11), anti-CD147 FITC (clone HIM6), anti-CD39 APC (clone TU66), CTLA-4 PE (clone BNI3) (BD Biosciences), anti-FoxP3 PE (clone PCH101), anti-CD127 PE-Cy7 (clone eBioRDR5), anti-IL-35 PE (ebic6), anti-PD1 PE (clone eBioJ105) (eBiosciences), anti-TGFβ PE (clone TB21; Cedarlene). Growth medium was prepared using RPMI-1640 (Mediatech, Inc) supplemented with 2mM Glutamine (Gemini Bio-Products), 20mM HEPES (Mediatech, Inc), 1% penicillin/streptomycin (Gemini Bio-Products), and 10% inactivated human serum (Gemini Bio Products). Recombinant human IL2 (rhIL2) was obtained from R&D Systems, Inc. Staining medium was prepared using 2% fetal bovine serum (Gemini Bio Products) in PBS.

### T-cell phenotypic characterization

Freshly thawed or CMV-stimulated PBMC were washed in staining medium and incubated for 30 min at 4°C with surface markers. For intracellular Granzyme B, TGFβ, IL-35 and IL-10 staining, PBMC were then permeabilized and fixed (Cytofix/Cytoperm; BD Biosciences) for 20 min at 4°C, washed and incubated with the appropriate mAbs. For intranuclear FoxP3, cells were permeabilized and fixed with IC Fixation and permeabilization buffer (eBiosciences) for 1 hour at 4°C, washed and incubated with the mAb. At the completion of the staining procedure, cells were fixed in 2% paraformaldehyde (Electron Microscopy Sciences) in PBS and data were acquired with Guava 8HT (Millipore) or Gallios (Beckman Coulter). Results were analyzed with FlowJo (Treestar) or Kaluza (Beckman Coulter).

### Cell sorting of CD3+CD27-CD28- or CD4+CD27-CD28- cells

Thawed PBMC were stained for CD4, CD3, CD27 and CD28 and sorted using MoFlo 70XDP (Beckman Coulter) and Summit Software. The average purity of cell sorts was 96.22%.

### Lymphocyte proliferation and expansion assays

PBMC at ≤10^6^ cells/mL in growth medium were incubated in quadruplicate wells of 96-well microplates (Corning) at 100μL/well in the presence of 100 μL of inactivated CMV-infected cell lysate, inactivated varicella zoster virus (VZV)-infected cell lysate, *C*. *albicans* antigen (Greer), HIV inactivated virion [26], the appropriate mock-infected cell control antigens and phytohemagglutinin (PHA; Sigma) positive control. After 6 days of antigenic or mitogenic stimulation at 37°C, in a 5% CO_2_ and 95% H_2_O atmosphere, cells were pulsed with 1 μCi of ^3^H-thymidine and harvested 6 h later onto Unifilter plates (Perkin Elmer). Radioactivity gathered on the filters was counted in scintillation fluid (Perkin Elmer) with a microplate scintillation counter (Packard). Results were expressed in median counts per minutes (cpm) of the quadruplicate wells. Inhibition was measured on samples that showed ≥3-fold increase of median cpm in antigen- compared with mock-stimulated wells.

Treg expansion with rhIL2 (30ng/mL) was carried out in 96-well microtiter plates as described above for up to 15 days, during which rhIL2 and growth medium were replenished twice weekly. At the end of the expansion, viable cells were counted with a Guava 8HT instrument.

### Statistical analysis

Two-tailed *t* tests were used for statistical analysis in Prism 6 (GraphPad) and a p value <0.0.5 was considered significant. Distribution of results was checked for normality and parametric tests were applied to normally distributed data. Log transformation was applied when indicated to achieve normal distribution of the data. Nonparametric tests were used for data with skewed distribution.

## Results

### Magnitude and phenotypic characterization of circulating CD4+CD27-CD28- T cells of CMV-infected compared with uninfected individuals

**Fig 1:**
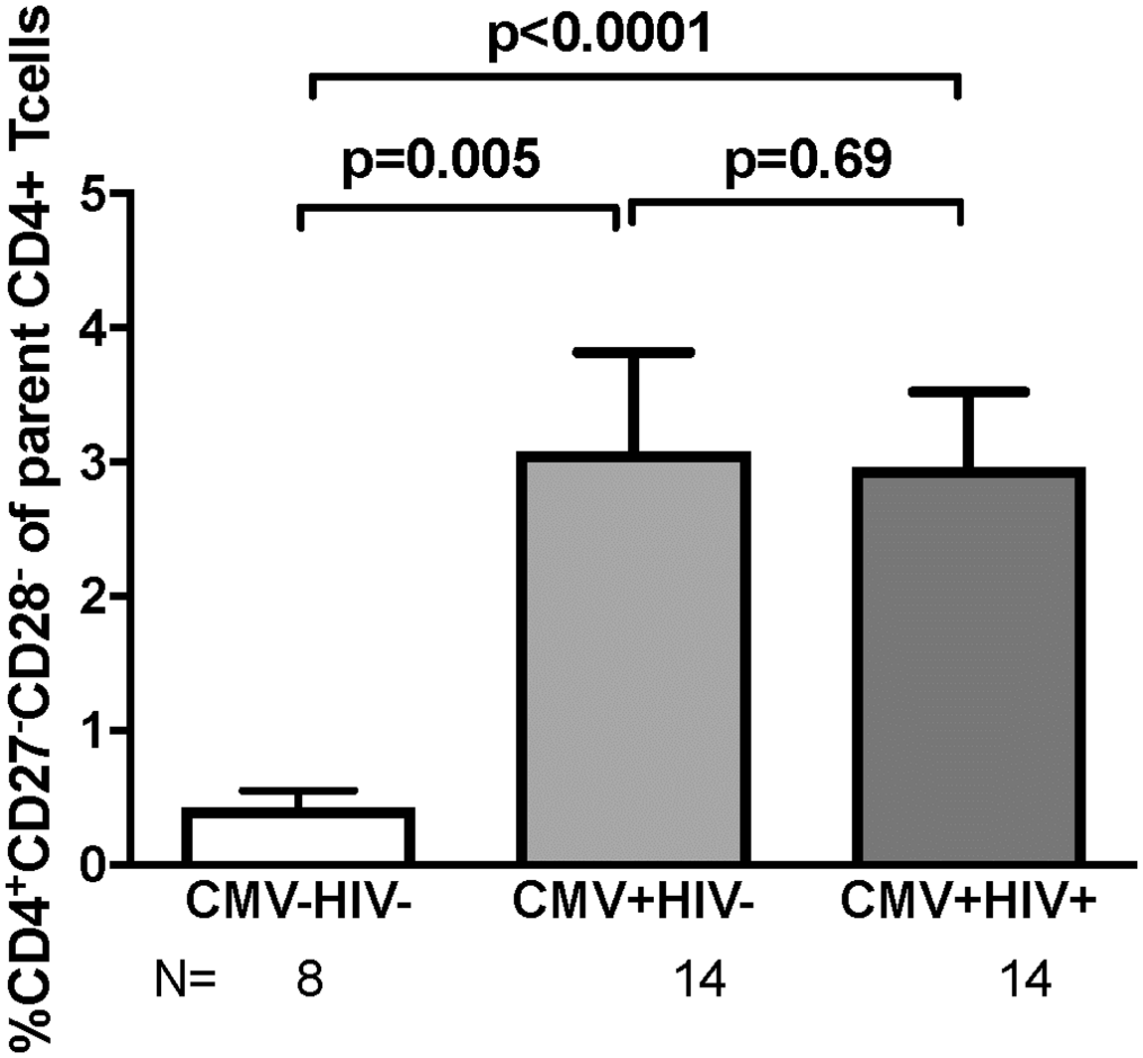
Frequency of CD4+CD27-CD28- T cells in peripheral blood of CMV-seronegative and CMV-seropositive with or without HIV co-infection. Data were derived from 36 donors. Bars indicate means and standard errors. N indicates the number of subjects in each group. P values were calculated by unpaired Student’s t test using log-transformed values.

To determine the effect of CMV infection on the magnitude of the circulating CD4+CD27-CD28- T cells, we compared PBMC from 8 CMV-seronegative (CMV-neg) HIV-seronegative (HIV-neg) blood donors, 14 CMV-seropositive (CMV-pos) HIV-neg blood donors or young adult volunteers and 14 CMV-pos HIV-seropositive (HIV-pos) individuals (Fig 1). The data showed significantly higher CD4+CD27-CD28-% in PBMC from CMV-pos donors regardless of HIV status compared with CMV-neg donors (means of ≥3.0% and 0.4%, respectively, p≤0.005; Fig 1).

**Fig 2:**
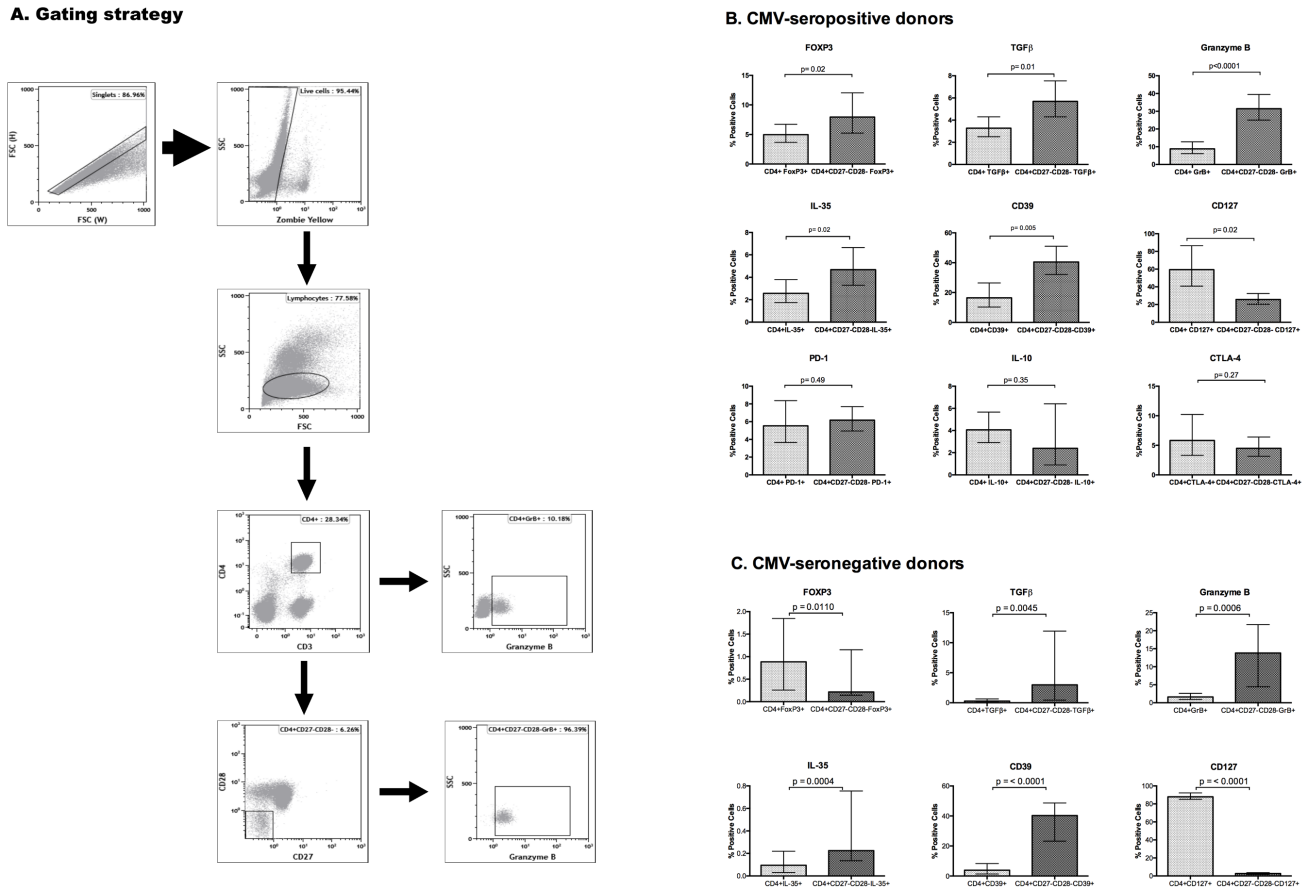
Phenotypic characterization of peripheral blood CD4+CD27-CD28- T cells of CMV-seropositive donors (B) and CMV-seronegative donors (C). **Panel A** shows the gating strategy. For **panels B** and **C** the data for each marker were derived from 7 to 20 CMV-seropositive or CMV-seronegative individuals per marker. The bars represent geometric means and 95% confidence intervals of the frequency of each marker in the entire CD4+ T cell population (light bars) and in the CD4+CD27-CD28- T cells only (dark bars). The marker studied is indicated in the title of each graph. P values were calculated by paired Student’s t test using log-transformed values.

Next, we compared Treg-defining phenotypic characteristics on circulating CD4+CD27-CD28- T cells and their parent CD4+ T cell populations in CMV-pos (HIV-pos or HIV-neg) participants (Fig 2). Using the gating strategy shown in Fig 2A, we show in Fig 2B that compared with the parent CD4+ T cells, significantly higher proportions of CD4+27-28- T cells from CMV-pos donors expressed FOXP3, TGFβ, GrB, IL-35 and CD39, but similar levels of IL-10 and CTLA-4. The CD4+CD27-CD28- subset also expressed higher levels of the Treg activation marker CD147. CD127 expression, which is usually low or absent on Tregs was significantly lower on the CD4+CD27-CD28- subset compared with the total CD4+ peripheral blood parent population. Fig 2C shows that CD4+CD27-CD28- peripheral blood T cells of CMV-neg individuals also expressed higher levels of TGFβ, GrB, IL-35 and CD39 and lower levels of CD127 compared with their parent total CD4+ T cells. In contrast to CMV-pos, the CMV-neg peripheral blood CD4+CD27-CD28- T cells had lower FOXP3 expression compared with the parent total CD4+ T cell population.

We also compared the ability of peripheral blood CD4+CD27-CD28- T cells from CMV-pos and CMV-neg individuals to expand ex vivo in the presence of rhIL2. Sorted CD4+CD27-CD28- T cells from 5 CMV-pos individuals underwent 5- to 24-fold increases in number over a period of 15 days (S1 Fig), whereas the cells from 3 CMV-neg individuals died during the first week of culture.

### Phenotypic and functional comparison of CD4+CD27-CD28- peripheral blood Treg-like cells with ex vivo CMV-restimulated CD4+CD27-CD28- Tregs

**Fig 3:**
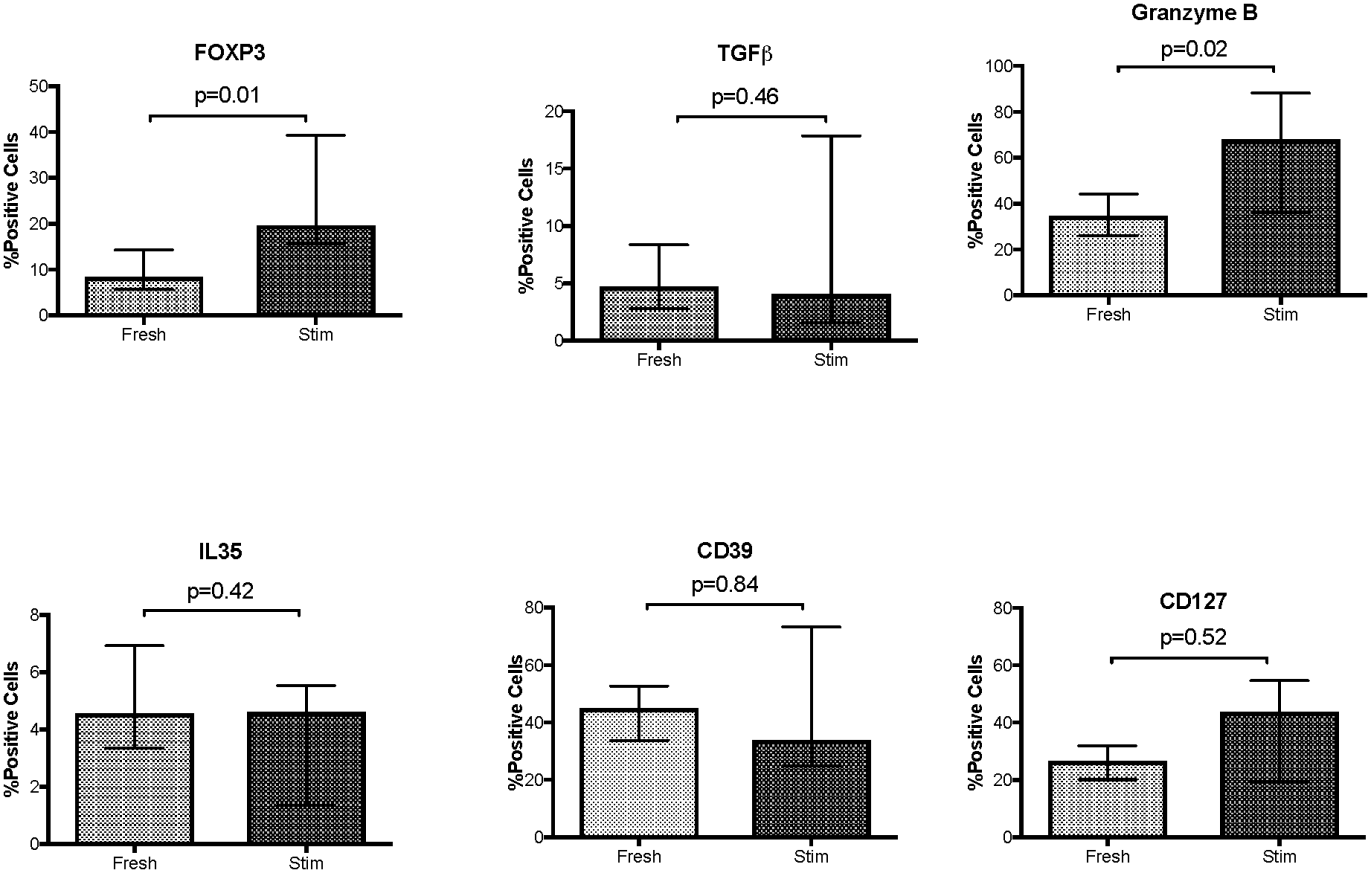
Phenotypic comparison of peripheral blood CD4+CD27-CD28- (Fresh) with *ex vivo* CMV-restimulated CD4+CD27-CD28- (Stim) T cells from CMV-seropositive donors. The data were derived from 4 to 21 donors per marker. Bars represent medians and interquartile ranges of the markers shown in the title of each graph. P values were calculated by Mann Whitney test for nonparametric distributions. S2 Fig shows the phenotypic characteristics of mock-stimulated PBMC from CMV-seropositive donors.

We previously showed that after 6 days of *ex vivo* restimulation of PBMC with CMV antigen high proportions of CD4+CD27-CD28- T cells in the PBMC cultures were associated with lower PBMC proliferations [15]. We further characterized the function and phenotype of the *ex vivo* CMV-stimulated CD4+CD27-CD28- T cells and found that they fulfilled the Treg definition [16], while mock-stimulated CD4+CD27-CD28- T cells expressed up to 10-fold lower levels of these markers (S2 Fig). Hence, we considered the *ex vivo* CMV-stimulated CD4+CD27-CD28- T cells a gold standard against which to compare the expression of Treg markers on the circulating CD4+CD27-CD28- T cells of CMV-pos donors (Fig 3). The two populations were similar with respect to TGFβ, IL35, CD39 and CD127 expression. However, significantly higher proportions of *ex vivo* CMV-restimulated CD4+CD27-CD28- Tregs expressed FOXP3 and GrB compared with peripheral blood CD4+CD27-CD28- Treg-like cells.

**Fig 4:**
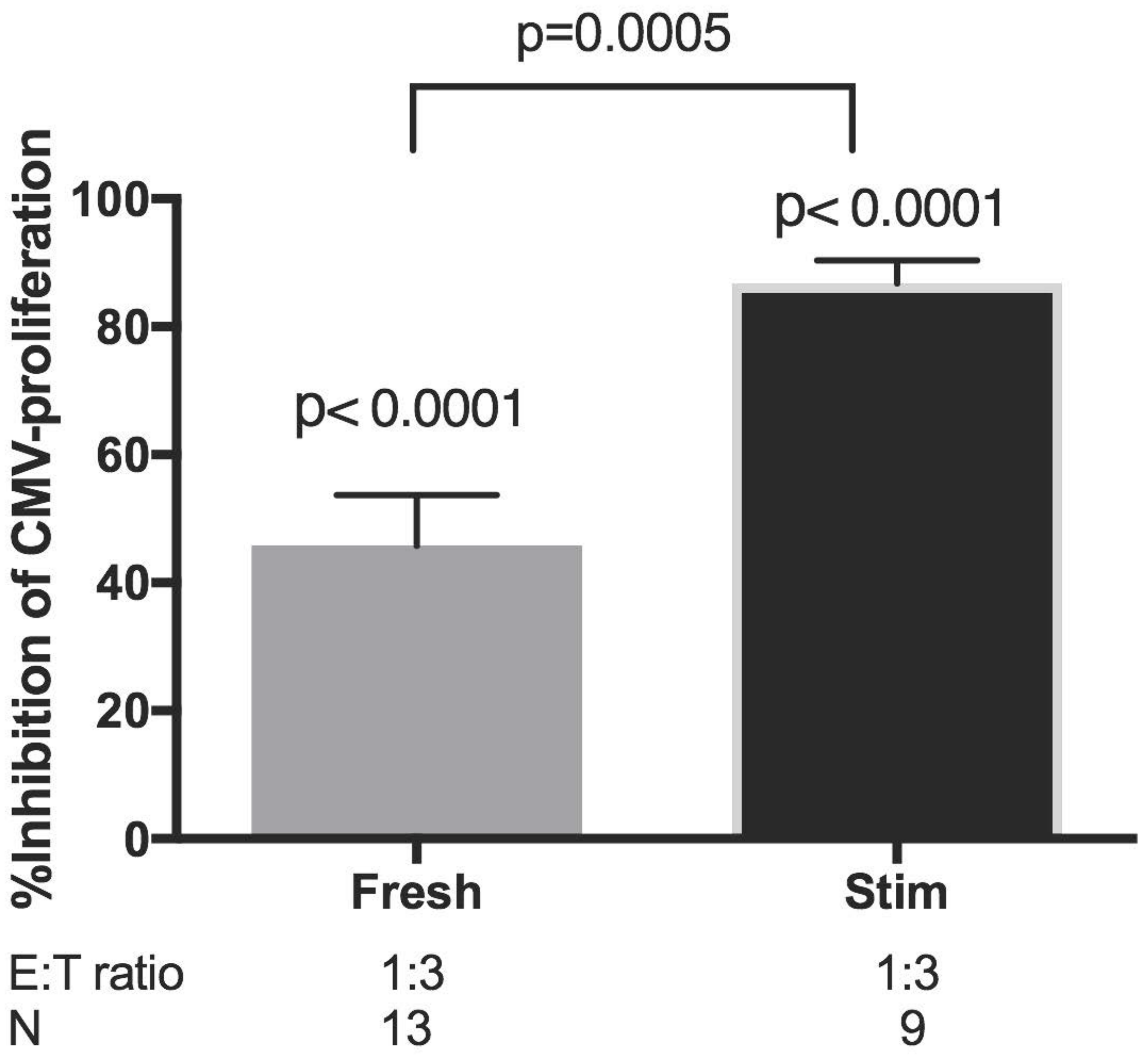
Functional evaluation of peripheral blood CD4+CD27-CD28- (Fresh) with *ex vivo* CMV-restimulated CD4+CD27-CD28- (Stim) T cells from CMV-seropositive donors. Bars represent means and SEM of % inhibition of proliferation calculated by the following formula: [(median CPM of CMV-stimulated wells) -(median CPM of CMV-stimulated + Treg wells)]/(median CPM of CMV-stimulated wells) x 100. E:T = Treg to autologous PBMC ratio. N = number of donors. P values above each column were calculated by single sample T test compared with 0% inhibition. P value between columns was calculated by unpaired T test. S3 Fig shows the same data presented in absolute CPM.

The functional comparison of peripheral blood CD4+CD27-CD28- T cells with CMV-stimulated CD4+CD27-CD28- Tregs used inhibition of LPA to maintain consistence with our previous studies. Peripheral blood CD4+CD27-CD28- T cells inhibited proliferation of autologous PBMC by an average of 47% at an Effector:Target (E:T) ratio of 1:3. CD4+CD27-CD28- Tregs generated by *ex vivo* CMV-restimulation inhibited by 87% the proliferation of autologous PBMC (Fig 4 and S3 Fig).

### Breadth of the regulatory function of peripheral blood CD4+CD27-CD28- Treg

**Fig 5:**
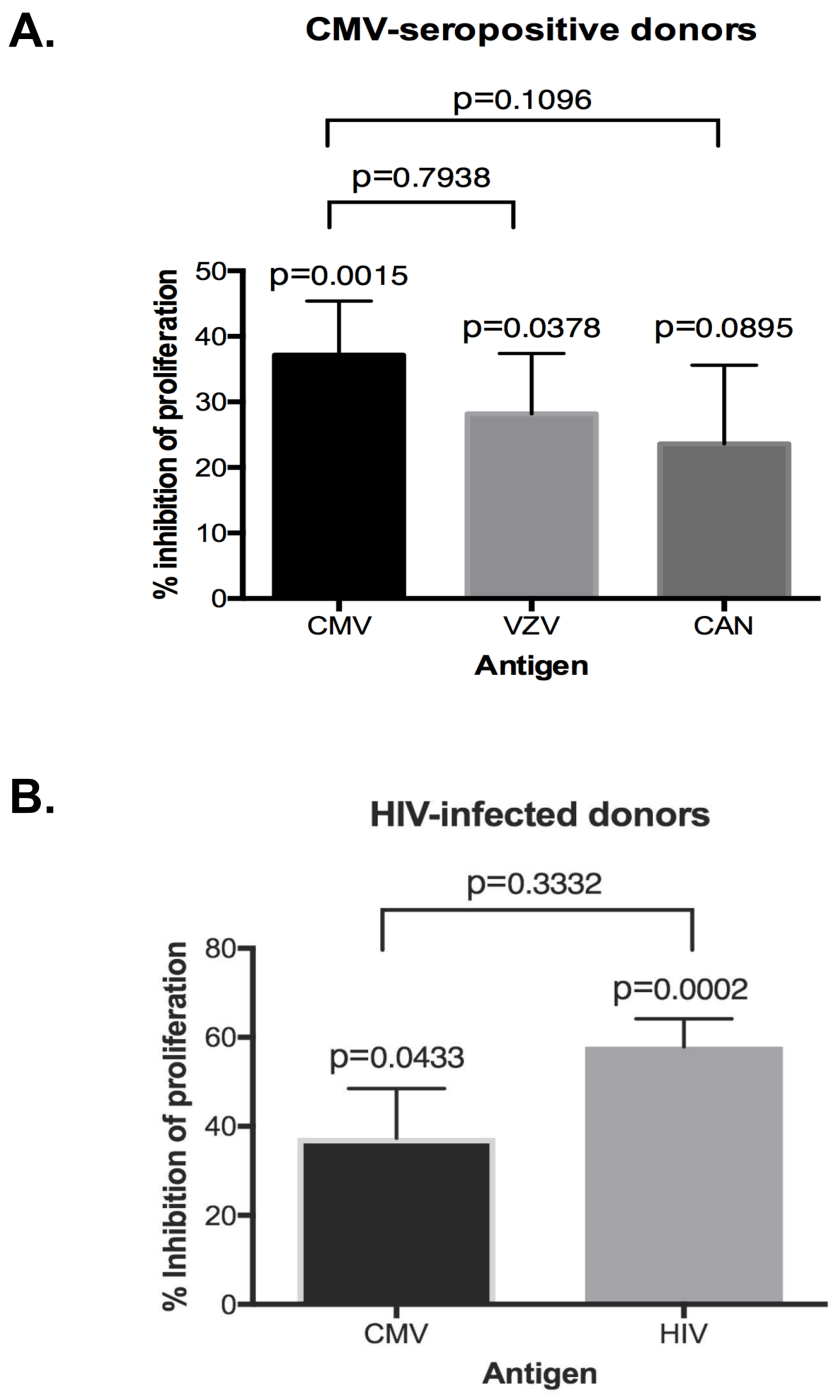
Breadth of inhibitory activity of peripheral blood CD4+CD27-CD28- Treg from CMV-seropositive donors. **Panel A** shows a comparison of CMV-), VZV- and candida-specific proliferation inhibition using sorted peripheral blood CD4+CD27-CD28- Treg and autologous PBMC from 5 to 10 donors/antigen at effector to target ratios = 1:3. **Panel B** shows inhibition of proliferation of PBMC from 6 HIV-pos CMV-pos donors stimulated with CMV or HIV by autologous peripheral blood CD3+CD27-CD28- Treg at an effector to target ratio = 1:3. Inhibition of proliferation was calculated by the following formula: [(median CPM of antigen-stimulated wells) -(median CPM of antigen-stimulated + Treg wells)]/(median CPM of antigen-stimulated wells) x 100. P values above each column were calculated by single sample T test compared with 0% inhibition. P values between columns were calculated by unpaired T test. S3 Fig shows the same data presented in absolute CPM.

We previously showed that *ex vivo* CMV-stimulated CD4+CD27-CD28- Tregs inhibited candida- and VZV *ex vivo* restimulated autologous PBMC in addition to CMV [16]. Candida and VZV were chosen because virtually all adults in the United States have been exposed to these antigens. In the current study, peripheral blood CD4+CD27-CD28- Tregs from 10 CMV-pos HIV-neg donors were added at a ratio of 1:3 to autologous PBMC in the presence of candida, VZV or CMV antigens. Proliferation measured after 6 days of culture was significantly inhibited when stimulated by VZV (mean = 28%; p = 0.04, one sample T test; Fig 5A and S3A Fig) at levels comparable with CMV (mean = 37%; p = 0.002; Fig 5A). Inhibition of candida-stimulated proliferation did not reach statistical significance (mean = 24%; p = 0.09; Fig 5A and S4A Fig). In contrast, CD4+CD27-CD28- T cells sorted from CMV-neg donors did not inhibit autologous PBMC proliferation in response to candida or VZV stimulation (S4B Fig).

Peripheral blood CD3+CD27-CD28- T cells were sorted from CMV-pos HIV-pos individuals for inhibition assays. We chose to sort CD3+CD27-CD28- T cells from these donors, because of the paucity of CD4+CD27-CD28- T cells and based on our previous study that showed that CD8+ T cells express FOXP3 and supplant the diminished number of CD4+ Tregs in HIV-infected individuals [15]. Moreover, we showed a significant association between the proportions of *ex vivo* CMV-stimulated cytotoxic CD8+ T cells and CMV end-organ disease or death in HIV-infected individuals independent of CMV blood viral load at the time when the PBMC were obtained [22]. The circulating CD3+CD27-CD28- T cells from HIV-pos CMV-pos donors inhibited proliferation of autologous PBMC *ex vivo* restimulated with inactivated HIV (mean = 58%, p = 0.0003; Fig 5B and S4C Fig) or CMV antigens (mean = 37%, p = 0.048; Fig 5B and S4C Fig) at an E:T ratio of 1:3.

## Discussion

Our data demonstrate that circulating CD4+CD28-CD27- T cells of CMV-pos donors have functional and phenotypic Treg characteristics, whereas circulating CD4+CD27-CD28- T cells of CMV-neg donors share selected phenotypic but not functional characteristics with those of CMV-pos donors. The phenotypic characterization used a broad panel of Treg markers, including FOXP3, which is a transcription factor that activates the regulatory program in T cells and is therefore considered the Treg hallmark; CD39, IL-35 and granzyme B, which are functional mediators of the Treg inhibitory activity; and TGFβ, which has both Treg activating and effector functions. Another Treg phenotypic characteristic found in the CD4+CD27-CD28- peripheral blood Treg was the low expression of CD127. Although many of these markers were shared by CMV-pos and CMV-neg donors, FOXP3 was found only in the circulating CD4+CD27-CD28- of CMV-pos donors. Moreover, only peripheral blood CD4+CD27-CD28- T cells from CMV-pos individuals proliferated in culture in the presence of rhIL2 and inhibited *ex vivo* proliferation of autologous PBMC. Taken together, these data indicate that the CD4+CD27-CD28- T cells of CMV-pos donors include Tregs, but those of CMV-neg individuals do not.

To verify the presence of Treg among the peripheral blood CD4+CD27-CD28- T cells, we used functional assays, namely inhibition of autologous PBMC proliferation. These assays showed that purified peripheral blood CD4+CD27-CD28- T cells from CMV-pos donors significantly and reproducibly decreased autologous PBMC proliferation not only in response to *ex vivo* CMV stimulation but also to VZV or candida stimulation. In contrast, circulating CD4+CD27-CD28- T cells of CMV-neg donors did not inhibit autologous proliferation. The functional difference between CMV-pos and CMV-neg peripheral blood CD4+CD27-CD28- T cells was in accordance with the lower expression of FOXP3 in the CMV-neg donor cells, emphasizing the importance of FOXP3 as a Treg marker. Moreover, the fact that only CD4+CD27-CD28- T cells from CMV-pos donors inhibited proliferation suggests that these Tregs originally generated in response to CMV, inhibited candida and VZV proliferation through cross-reactivity or lack of specificity.

It is important to note the similarities and differences between the peripheral blood CD4+CD27-CD28- T cells of CMV-pos donors and those generated *ex vivo* through CMV antigenic stimulation. Both CD4+CD27-CD28- T-cell populations expressed Treg phenotypic markers and inhibited proliferation of autologous PBMC in response to CMV, VZV and candida antigenic stimulation. However, compared with circulating CD4+CD27-CD28- T cells, *ex vivo* expanded CD4+CD27-CD28- T cells contained higher proportions of FOXP3+ and GrB+ cells and had greater capacity to inhibit CMV-stimulated proliferation of autologous PBMC, reinforcing the notion that the proportion of FOXP3+ cells provides a good indication of the presence of Tregs among T cells that phenotypically might be considered differentiated effectors.

Whether inducible Tregs evolve from natural Tregs or conventional T cells is the topic of current investigations [27–30]. A recent study showed that 12% of T-cell receptors were shared by Tregs and conventional effector T cells at the DNA level suggesting a common progenitor cell [31]. Tregs also tend to express markers of the effectors that they target. For example, Tregs inhibiting Th1 responses express CXCR3, Tbet and sometimes IFNγ, whereas those inhibiting Th17 responses express CCR6, RORγt and sometimes IL17 [32]. The common phenotypic characteristics between differentiated effectors and the Tregs described in our study support the notion that inducible Tregs evolve in parallel or sequentially with the differentiated effectors. The corollary of this observation is that the frequency or magnitude of CMV stimulation during reactivations, which is likely to be associated with the frequency of differentiated effector T cells, also may also dictate the magnitude of the CMV-induced Treg population. This hypothesis needs to be tested in additional studies, because its clinical implication is that prevention of CMV reactivations may substantially decrease the CMV-associated immune suppression of old adults and immune compromised hosts.

It may also be clinically significant that CD3+CD27-CD28- T cells sorted from the peripheral blood of CMV-pos HIV-infected individuals inhibited cell-mediated immune responses to ex vivo HIV antigenic restimulation. In these experiments, we used CD3+CD27-CD28- T cells because of the paucity of CD4+ T cells in HIV-infected individuals and because our previous studies suggested that CD8+ T cells play an important regulatory role in HIV-infected donors [15, 22], which is in agreement with the findings of other investigators who also showed that CD8+ inducible Tregs have the same function as their CD4+ counterparts [33–40]. Moreover, in CMV-pos old adults both CD4+CD28- and CD8+CD28- T cell numbers positively correlated with immune-suppression [41–43]. In the case of HIV infection, the effect of CMV Tregs on HIV-specific immunity may explain the strong association between CMV viremia and death of HIV-infected individuals that is not explained by CMV end-organ disease and is not associated with HIV viremia [2].

Based on these data, we propose that CMV reactivations generate both effector and regulatory responses that imprint the immune system of the host. The Tregs associated with CMV infection have a cross reactive down regulatory effect on the cell-mediated immune responses against other antigens leading to a generalized regulatory environment in the host that may not be apparent in immune competent hosts, but it may have biological significance in immune compromised hosts. Future studies are needed to determine if the frequencies of peripheral blood CD4+CD27-CD28- or CD8+CD27-CD28- Tregs in CMV-pos hosts increase with the number, duration and/or magnitude of viral replication during CMV reactivations.

## Supporting information

S1 Fig CD4+CD27-CD28- regulatory T cells from CMV-pos donors expand in vitro in the presence of rhIL2. Data were derived from 5 CMV-pos donors. CD4+CD27-CD28- T cells were sorted and cultured in growth medium with 30ng/mL rhIL2. Cells expanded 5- to 25-fold after 15 days of culture.
(PDF)

S2 Fig Culture of PBMC from CMV-pos individuals does not select for CD4+CD27-CD28- with Treg characteristics. Data were derived from 4 donors. PBMC were stimulated with uninfected human lung fibroblast lysate for 6 days, washed, stained and analyzed with the Gallios instrument and Kaluza software. Bars indicate means and SEM.
(PDF)

S3 Fig Functional evaluation of peripheral blood CD4+CD27-CD28- (Circulating) with ex vivo CMV-restimulated CD4+CD27-CD28- (CMV-stimulated) T cells from CMV-seropositive donors. Data were derived from 9 CMV-pos donors in panel A and 13 CMV-pos donors in panel B. PBMC were stimulated with CMV lysate at 66,000 cells/well for 6 days with and without the addition 33,000 autologous CD4+CD27-CD28- Treg. Cells were pulsed with 3HThy for the last 6 h of incubation, harvested and proliferation was measured by 3H incorporation.
(PDF)

S4 Fig Only CD4+CD27-CD28- from CMV-pos individuals are Treg. Data were derived from 10 CMV-pos donors in panel A; 4 CMV-neg in panel B and 6 HIV-pos CMVpos in panel C. 66,000 PBMC per well were incubated in triplicate or quadruplicate wells for 6 days with the antigens indicated on each graph, with and without autologous CD4+CD27-CD28- (panels A and B) or CD3+CD27-CD28- panel C).
(PDF)
