# Supplementary material for: Cytomegalovirus infection in HIV-infected and uninfected individuals is characterized by circulating regulatory T cells of unconstrained antigenic specificity
Source: PLoS One. 2017 Jul 6;12(7):e0180691. doi: 10.1371/journal.pone.0180691 (PMC5500357; doi:10.1371/journal.pone.0180691)
Supplement: S1 Fig — Data were derived from 5 CMV-pos donors. CD4+CD27-CD28- T cells were sorted and cultured in growth medium with 30ng/mL rhIL2. Cells expanded 5- to 25-fold after 15 days of culture. (PDF) [file pone.0180691.s001.pdf]

CD4+CD27-CD28- Cell Expansion with IL-2 (30ng/mL)

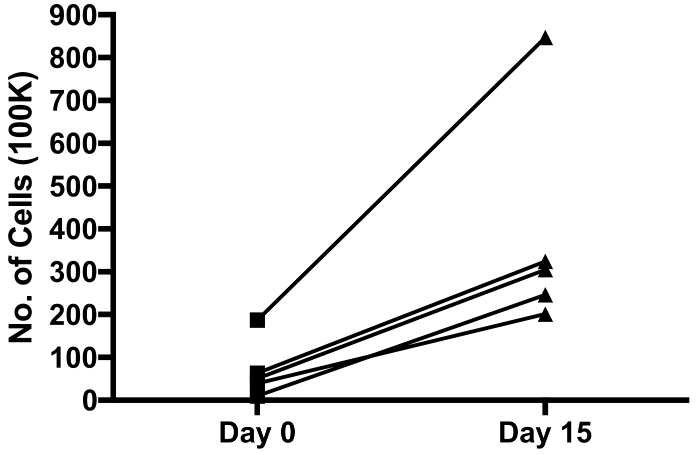

S1 Fig. CD4+CD27-CD28- regulatory T cells from CMV-pos donors expand in vitro in the presence of rhIL2. Data were derived from 5 CMV-pos donors. CD4+CD27-CD28- T cells were sorted and cultured in growth medium with 30ng/mL rhIL2. Cells expanded 5- to 25-fold after 15 days of culture.
