# Supplementary material for: Cytomegalovirus infection in HIV-infected and uninfected individuals is characterized by circulating regulatory T cells of unconstrained antigenic specificity
Source: PLoS One. 2017 Jul 6;12(7):e0180691. doi: 10.1371/journal.pone.0180691 (PMC5500357; doi:10.1371/journal.pone.0180691)
Supplement: S2 Fig — Data were derived from 4 donors. PBMC were stimulated with uninfected human lung fibroblast lysate for 6 days, washed, stained and analyzed with the Gallios instrument and Kaluza software. Bars indicate means and SEM. (PDF) [file pone.0180691.s002.pdf]

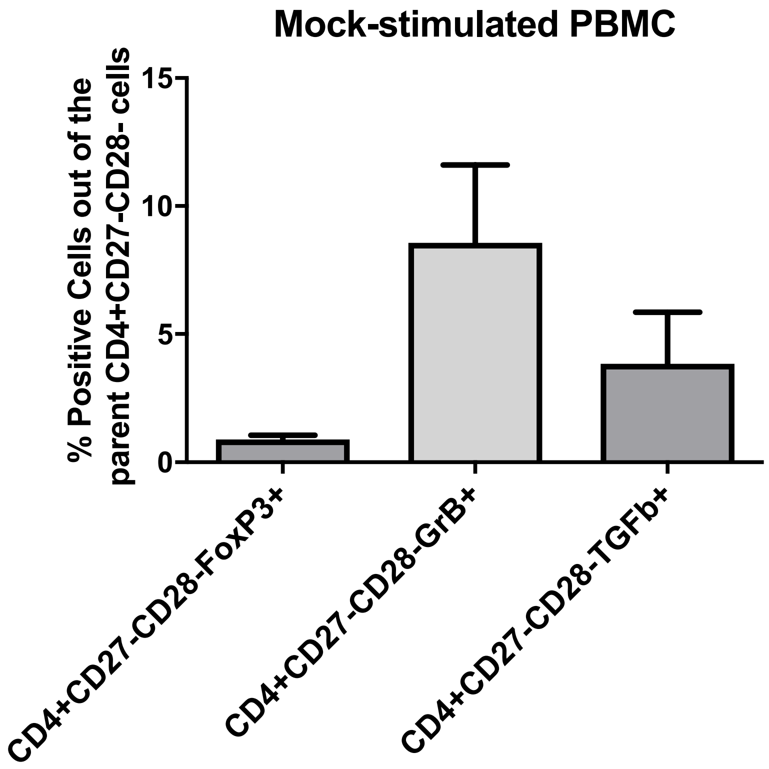

S2 Fig. Culture of PBMC from CMV-pos individuals does not select for CD4+CD27-CD28- with Treg characteristics. Data were derived from 4 donors. PBMC were stimulated with uninfected human lung fibroblast lysate for 6 days, washed, stained and analyzed with the Gallios instrument and Kaluza software. Bars indicate means and SEM.
