# Supplementary material for: Cytomegalovirus infection in HIV-infected and uninfected individuals is characterized by circulating regulatory T cells of unconstrained antigenic specificity
Source: PLoS One. 2017 Jul 6;12(7):e0180691. doi: 10.1371/journal.pone.0180691 (PMC5500357; doi:10.1371/journal.pone.0180691)
Supplement: S3 Fig — Data were derived from 9 CMV-pos donors in panel A and 13 CMV-pos donors in panel B. PBMC were stimulated with CMV lysate at 66,000 cells/well for 6 days with and without the addition 33,000 autologous CD4+CD27-CD28- Treg. Cells were pulsed with 3HThy for the last 6 h of incubation, harvested and proliferation was measured by 3H incorporation. (PDF) [file pone.0180691.s003.pdf]

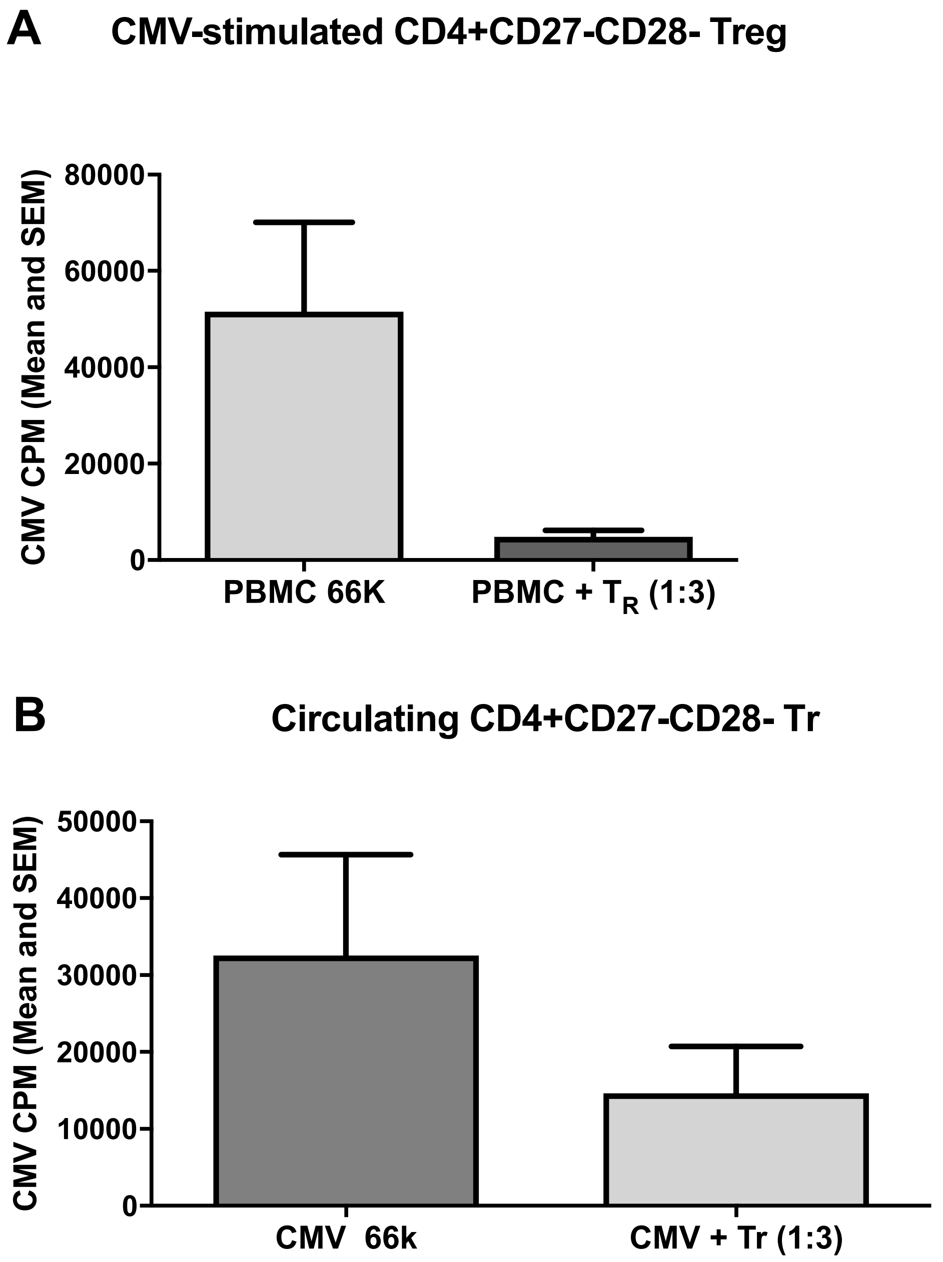

S3 Fig. Functional evaluation of peripheral blood CD4+CD27-CD28- (Circulating) with *ex vivo* CMV-restimulated CD4+CD27-CD28- (CMV-stimulated) T cells from CMV-seropositive donors. Data were derived from 9 CMV-pos donors in panel **A** and 13 CMV-pos donors in panel **B**. PBMC were stimulated with CMV lysate at 66,000 cells/well for 6 days with and without the addition 33,000 autologous CD4+CD27-CD28- Treg. Cells were pulsed with ^{3}HThy for the last 6 h of incubation, harvested and proliferation was measured by ^{3}H incorporation.
