# Supplementary material for: Cytomegalovirus infection in HIV-infected and uninfected individuals is characterized by circulating regulatory T cells of unconstrained antigenic specificity
Source: PLoS One. 2017 Jul 6;12(7):e0180691. doi: 10.1371/journal.pone.0180691 (PMC5500357; doi:10.1371/journal.pone.0180691)
Supplement: S4 Fig — Data were derived from 10 CMV-pos donors in panel A; 4 CMV-neg in panel B and 6 HIV-pos CMVpos in panel C. 66,000 PBMC per well were incubated in triplicate or quadruplicate wells for 6 days with the antigens indicated on each graph, with and without autologous CD4+CD27-CD28- (panels A and B) or CD3+CD27-CD28- panel C). (PDF) [file pone.0180691.s004.pdf]

A

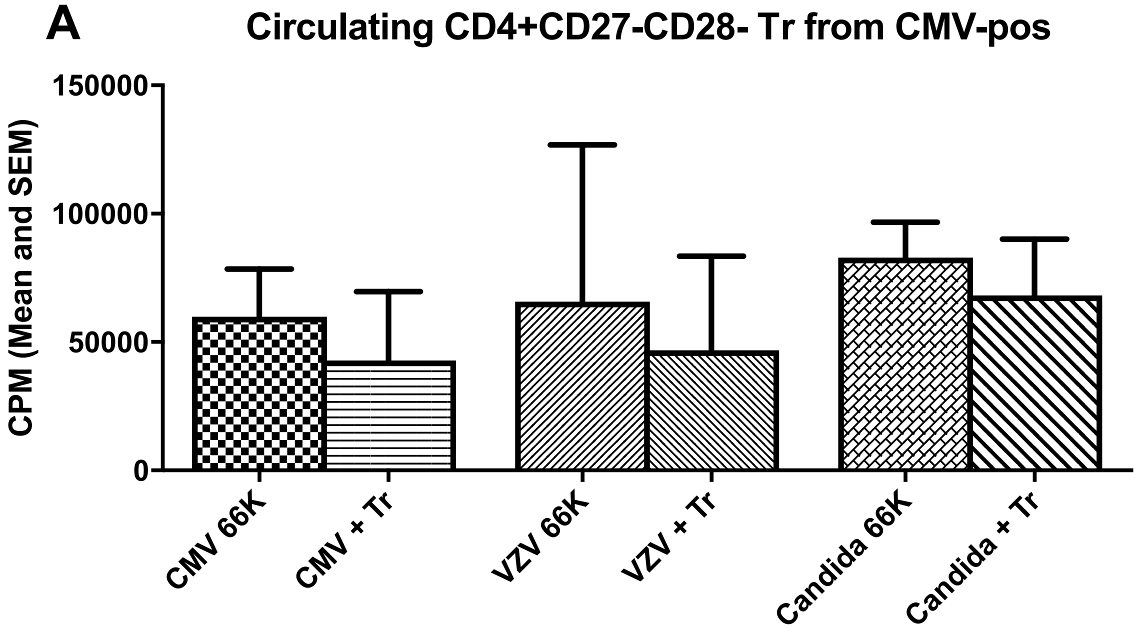
Circulating CD4+CD27-CD28- Tr from CMV-pos
CPM (Mean and SEM)
150000
100000
50000
0
CMV 66K
CMV + Tr
VZV 66K
VZV + Tr
Candida 66K
Candida + Tr

B

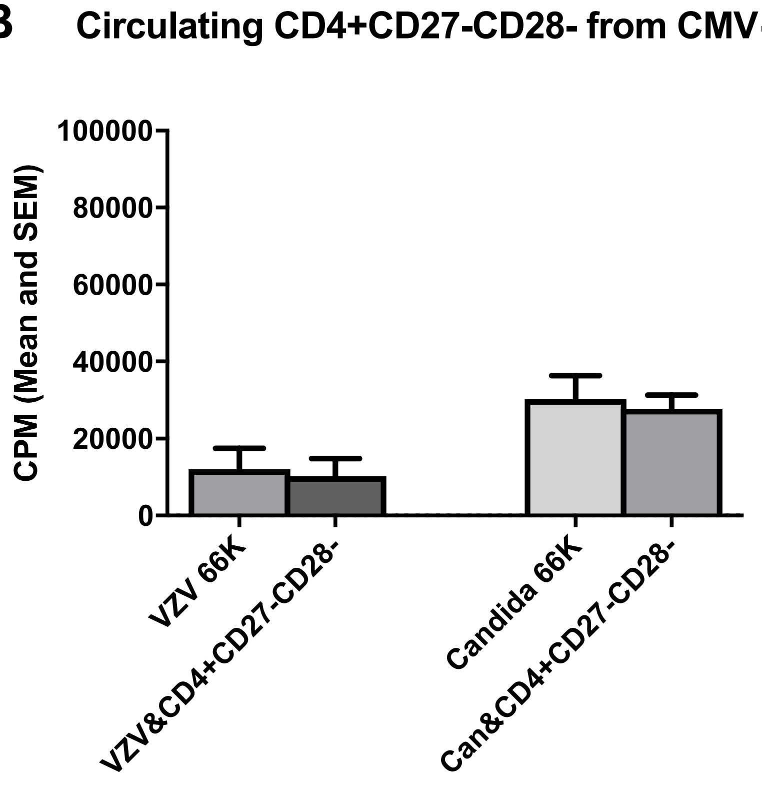
Circulating CD4+CD27-CD28- from CMV-neg
CPM (Mean and SEM)
100000
80000
60000
40000
20000
0
VZV 66K
VZV&CD4+CD27-CD28-
Candida 66K
Can&CD4+CD27-CD28-

C

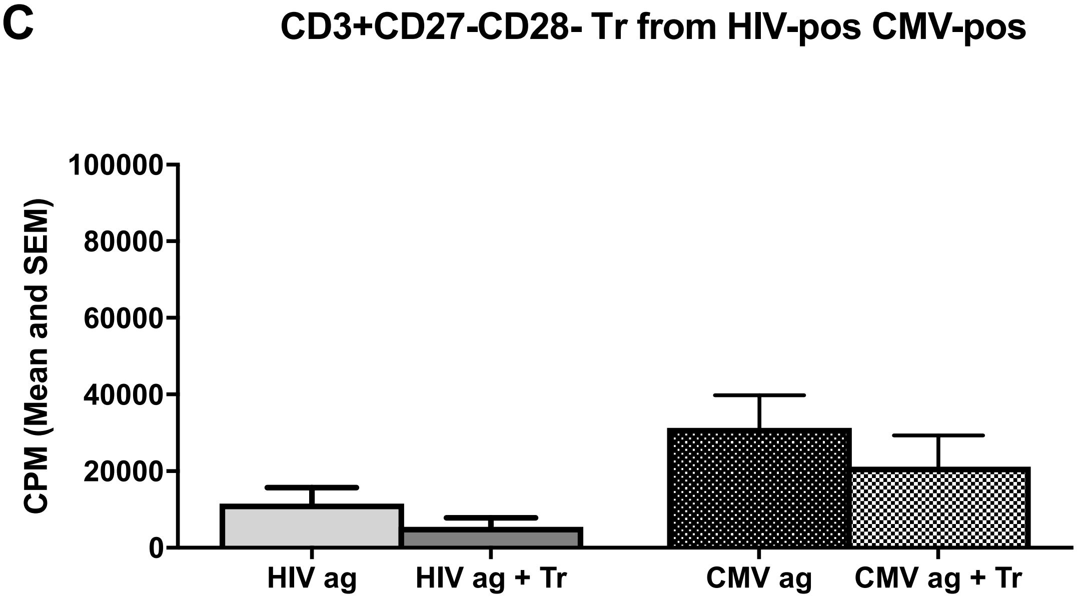

S4 Fig. Only CD4+CD27-CD28- from CMV-pos individuals are Treg. Data were derived from 10 CMVpos donors in panel **A**; 4 CMVneg in panel **B** and 6 HIVpos CMVpos in panel **C**. 66,000 PBMC per well were incubated in triplicate or quadruplicate wells for 6 days with the antigens indicated on each graph, with and without autologous CD4+CD27-CD28- (panels A and B) or CD3+CD27-CD28- panel C).
